# Large-scale radio propagation path loss measurements and predictions in the VHF and UHF bands

**DOI:** 10.1016/j.heliyon.2021.e07298

**Published:** 2021-06-15

**Authors:** Nasir Faruk, I.Y. Abdulrasheed, N.T. Surajudeen-Bakinde, Emmanuel Adetiba, A.A. Oloyede, Abubakar Abdulkarim, Olugbenga Sowande, Ayodele H. Ifijeh, Aderemi A. Atayero

**Affiliations:** a Department of Telecommunication Science University of Ilorin, Nigeria; b Department of Physics, Sule Lamido University, Kafin Hausa, Nigeria; c Department of Electrical and Electronics Engineering, University of Ilorin, Nigeria; d Department of Electrical and Information Engineering, Covenant University, Ota, Nigeria; e Department of Electrical Engineering, Ahmadu Bello University Zaria, Nigeria

**Keywords:** Path loss measurement, Kriging Interpolation Method, Prediction model, Radio propagation

## Abstract

For decades now, a lot of radio wave path loss propagation models have been developed for predictions across different environmental terrains. Amongst these models, empirical models are practically the most popular due to their ease of application. However, their prediction accuracies are not as high as required. Therefore, extensive path loss measurement data are needed to develop novel measurement-oriented path loss models with suitable correction factors for varied frequency, capturing both local terrain and clutter information, this have been found to be relatively expensive. In this paper, a large-scale radio propagation path loss measurement campaign was conducted across the VHF and UHF frequencies. A multi-transmitter propagation set-up was employed to measure the strengths of radio signals from seven broadcasting transmitters (operating at 89.30, 103.5, 203.25, 479.25, 615.25, 559.25 and 695.25 MHz respectively) at various locations covering a distance of 145.5 km within Nigerian urban environments. The measurement procedure deployed ensured that the data obtained strictly reflect the shadowing effects on radio signal propagation by filtering out the small-scale fading components. The paper also, examines the feasibilities of applying Kriging method to predict distanced-based path losses in the VHF and UHF bands. This method was introduced to minimize the cost of measurements, analysis and predictions of path losses in built-up propagation environments.

## 1 Introduction

Wireless systems and services have recently become highly indispensable in everyday activities of the human populace. They are crucial in our day-to-day activities as they are deployed at homes, business places, and places of worships, schools, hospitals, markets and many other places. They have essentially, become part of our lives. Furthermore, these days, systems such as the cellular mobile, television broadcasting, and public safety networks are important indices for measuring development. In wireless communication systems, electromagnetic signals (i.e. radio waves) are propagated through air and due to the unique features of this medium, this type of propagation is affected by the presence of terrain and clutters such as buildings, vehicular movements, girders, mountains and trees, obstructing the communication paths, resulting in signal reflection, attenuation and sometimes diffraction or scattering (Rappaport, 1996). Reflections of the radio waves occur when the signal interacts with objects whose dimensions are greater than the wavelength of the travelling wave, otherwise, scattering is experienced. Diffraction on the other hand, occurs, when the wave interacts with an object with irregular surfaces. Other effects are signal absorption, interference and refraction. All these are varied phenomenon that often resulted in signal fading. This may be a small-scale fading when the signal is propagated within a short duration and distance, resulting to rapid fluctuations of signal strength or a large-scale fading when it happens over a large distance, resulting to propagation losses. This large-scale fading is commonly refers to as path loss (Faruk et al., 2013b). Significant path loss propagation measurements have been conducted in different scenarios to study the propagation attenuation and characteristics (Igbinosa and Okpeki, 2019; Akinbolati and Ajewole, 2020). (Zheng et al., 2018) conducted field strength measurements across 16 channels to model signal fluctuation in a tunnel that is 100 m long. During the experiment, two CC2530 modules were used to collect 20 received signal strength values continuously at each position and averaged. This process was conducted for every 0.1 m from 0 to 25 m. In (Al-Samman et al., 2018), large-scale path loss propagation models were derived from indoor propagation measurements conducted on the ultrawideband and millimetre wave (mmWave) at 28 GHz and 38 GHz in-building communication. The indoor-to-indoor and also outdoor -to-indoor study of radio wave propagation for long-term evolution (LTE) broadband deployments in high speed railways in Spain is provided by (Zhang et al., 2017). In the work, extensive path loss propagation measurements were conducted across Sub 10 GHz and frequencies considered were 2.4, 2.6 and 5.7 GHz. In (de Carvalho et al., 2021) optimum path loss parameters were obtained using meta-heuristic optimization such as Cuckoo Search (CS) for LTE deployment. Similar work was conducted in cruise ship in (Mariscotti, 2011) to determine the signal attenuation and propagation loss parameters. (González-Palacio et al., 2021) shows how Support Vector Machine (SVM) is more accurate in predicting signal of WLAN network than simplified path loss lognormal shadow fading model.

Link budget analysis and path loss prediction are very essential ingredients in the design of any wireless communication network (Popoola et al., 2018b). The large scale path loss models, for prediction of signal power within very long distance, are statistical in nature, and used to describe the behavior of wireless channels, due to their inherent randomness (Mitra, 2009). Many propagation models (deterministic, analytical or empirical) have been proposed, developed (Zheng et al., 2018); and used for decades to predict path losses in different environmental terrains across various frequencies (Phillips et al., 2013). Amongst these models, the empirical path loss models are the most widely used in practice due to their simplicity and ease of application. Some popular and widely cited models are the Hata (1980); COST 231 (Faruk et al., 2014) and Egli models (Egli, 1957). Even though they are practically popular (Popoola and OSeni, 2014); (Faruk et al., 2013a), it has been found that most of these models do not reflect the dynamic variation of the signal level, thus, giving high prediction errors when tested in different terrain environments other than the ones they were initially built for (Jimoh et al., 2015b, Surajudeen-Bakinde et al., 2018, Faruk et al., 2017, Phillips et al., 2012, Jimoh et al., 2015a). Moreover, significant path loss data are needed to come-up with a robust empirical model with sets of correction factors, as measurements needed to be conducted in several environments and across bands, capturing both local terrain and clutter information, which have been found to be relatively expensive. Of recent, artificial intelligence methods have been deployed in either tuning the path loss system parameters or developing models that provide better prediction accuracy. It is worth noting the works by (Popoola et al., 2019, Faruk et al., 2019, Popoola et al., 2018a, Faruk et al., 2019a, Faruk et al., 2019b). Extensive surveys and meta-data analysis of various works that applied heuristic algorithms are provided in (Adebowale et al., 2021, Aragón-Zavala et al., 2021).

The need to develop a robust model means large scale path loss data have to be collected across many routes and various frequency bands. However, this can be tedious when considering the limitations of the existing measurement set-ups, validities of the instruments (equipment) deployed for path loss data collection, the cost and time constraints during the measurement campaigns. The existing and most widely used approach found in literatures (Zheng et al., 2018, Al-Samman et al., 2018, Mariscotti, 2011, Hata, 1980, Surajudeen-Bakinde et al., 2018) for setting-up the instruments, usually scans a specific frequency of interest and measurements are been conducted along specific routes. Therefore, if 7 frequencies and 3 routes will be considered for the experiments as in the case of this work, the experimental set-up and measurements have to be repeated (7 × 3) times which will eventually add costs apart from the time needed. These, therefore, necessitate the continued efforts for cost effective set-up and methodologies for large scale path loss propagation measurements that would enhance the development of novel measurement-oriented path loss models with suitable correction factors for varied frequencies.

Kriging Interpolation Method (KIM) is a geostatistical spatial interpolation technique that was introduced in (Krige, 1951) for mining exploration. This approach ordinarily, removes the need for knowledge regarding the specifics of the propagation parameters and also, minimizes large scale path loss data as required by the empirical models, even though, it requires some sample data be collected before interpolation can be made as contrast to a well-developed empirical model. There are quite some works that have applied KIM for different predictions, these include: quantification of beam vibration (Krishnan and Ganguli, 2021); Raster data projection (Meng, 2021); prediction of rock joint shear strength (Hasanipanah et al., 2021) and Wi-Fi RSS fingerprints (Kram et al., 2017). Recently, Hybrid Kriging and multilayer perceptron neural network technique was used to predict coverage prediction in cellular networks (Mezhoud et al., 2020). It is worthy-noting that, KIM method has not been widely applied to distance-based path loss predictions, particularly, in the VHF and UHF spectrum bands. The paper therefore, developed Kriging algorithm for distance-based path loss prediction in the VHF and UHF bands.

## 2 Materials and method

In this section, the description of the measurement procedure used during the path loss propagation measurements is presented. Furthermore, data pre-processing (filtering and normalization) process employed were also provided.

### 2.1 Measurement locations and transmitters details

In this study, two urban Nigerian cities were used for the path loss propagation measurements. The cities are: Ilorin and Osogbo with the coordinates of (8.5° N, 4.55° E) and (7.7667° N, 4.5667° E) respectively. The measurements were conducted in two phases. Phase I, was conducted in VHF bands in Ilorin metropolis while, phase II, in the UHF bands in Osogbo. The path loss propagation measurements campaign covers only the broadcasting frequencies on the VHF and UHF bands. This is done using a total of seven (7) transmitters. Three of the transmitters operate on the VHF bands, while, the remaining four operate on the UHF bands. The routes considered in both cities were characterized by a high number of diffraction and scattering. This is because the average distance between buildings ranges from 30m to 40m and they are mostly concentrated along the road used in taking the measurements. For the Ilorin campaign, electromagnetic field strength was measured across three predefined routes (i.e. routes 1 to 3 in this work). These routes are all within the metropolitan area of the state and are characterized as urban. The clutter covers mainly buildings which are distributed along the routes, moving vehicles and thick vegetations with undulating terrain elevation. The elevation along these routes ranges from 150m to 320 m. None Line of Sight (NLOS) propagation between the receiver and the transmitters is also the most dominant as there are lots of high rise buildings along the route. The distance covered and number of path losses data collected along these routes are: 13.5 km and 8934 for route 1, 10 km and 10,019 for route 2 and 9 km and 6758 for route 3. The three VHF broadcast transmitters are: the Unilorin, Harmony, and NTA transmitters. The operating frequencies of the transmitters are 89.30 MHz, 103.5 MHz and 203.25 MHz with coordinates 8° 29′ 21″ N, 4° 40′ 28″ E; 8° 21′ 56″ N, 4° 43′ 18″ E and 8° 25′ 55″ N, 4° 36’ 25” E respectively. A summary is provided in Table 1.

**Table 1:** Characteristics of the broadcast transmitters.

Transmitter	Location	Band	Coordinates		Centre Frequency (MHz)	Height (m)	Tx. Power (kW)
			Latitude	Longitude			
UNILORIN	Ilorin	VHF	8° 29′ 21″ N	4° 40′ 28″ E	89.30	100	1.0
HARMONY	Ilorin		8° 21′ 56″ N	4° 43′ 18″ E	103.5	125	7.0
NTA, ILORIN	Ilorin		8° 25′ 55″ N	4° 36′ 25″ E	203.25	185	2.4
NDTV	Ibokun	UHF	7° 46′ 32″ N	4° 43′ 14″ E	479.25	198	2.1
OSBC	Osogbo		7° 46′ 35″ N	4° 35′ 19″ E	559.25	340	3.5
NTA, IFE	Ile-Ife		7° 29′ 59″ N	4° 35′ 23″ E	615.25	167	3.2
NTA, OSOGBO	Osogbo		7° 44′ 01″ N	4° 31′ 14″ E	695.25	152	4.1

For the Osogbo measurements, four routes (i.e. routes 1 – 4b), were considered. All the routes are also categorized as urban, suburban and open area. The routes consist of cluttered buildings with heavy vehicular movements as the routes are along a dual carriage motorway. Diffraction and scattering are common occurrences along these routes because of the presence of thick plantations and high-rise buildings. The distance covered and number of samples collected along these routes are 30 km and 10,015 for route 1b, 25 km and 7235 for route 2b, 25 km and 26,323 for route 3b and 33 km and 2587 for route 4b. The four UHF broadcast transmitters used are NDTV, NTA Ile-Ife, OSBC and NTA Osogbo. They operate on frequencies 479.25 MHz, 615.25 MHz, 559.25 MHz and 695.25 MHz, respectively. Furthermore, all the transmitters were deployed at fixed locations, being that details about their coordinates, heights and power levels can be found in Table 1. The overall total of 145.5 km was covered and 71871 samples were collected. An average height of 1.5 m for the receiver is assumed across the routes for the period of the measurements, even though this is expected to undulate with elevation and depression. A summary is provided in Table 1.

### 2.2 Measurement setup

**Figure 1:**
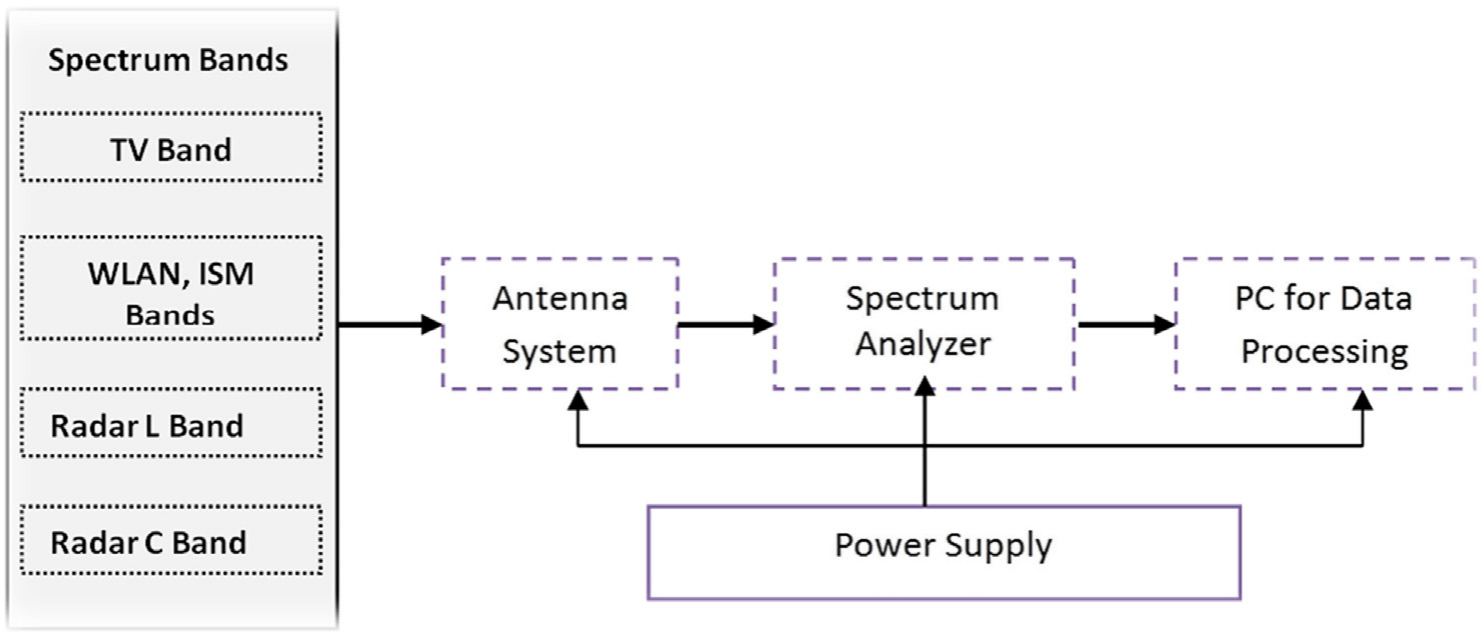
Path Loss Propagation Measurement Set-up framework.

**Figure 2:**
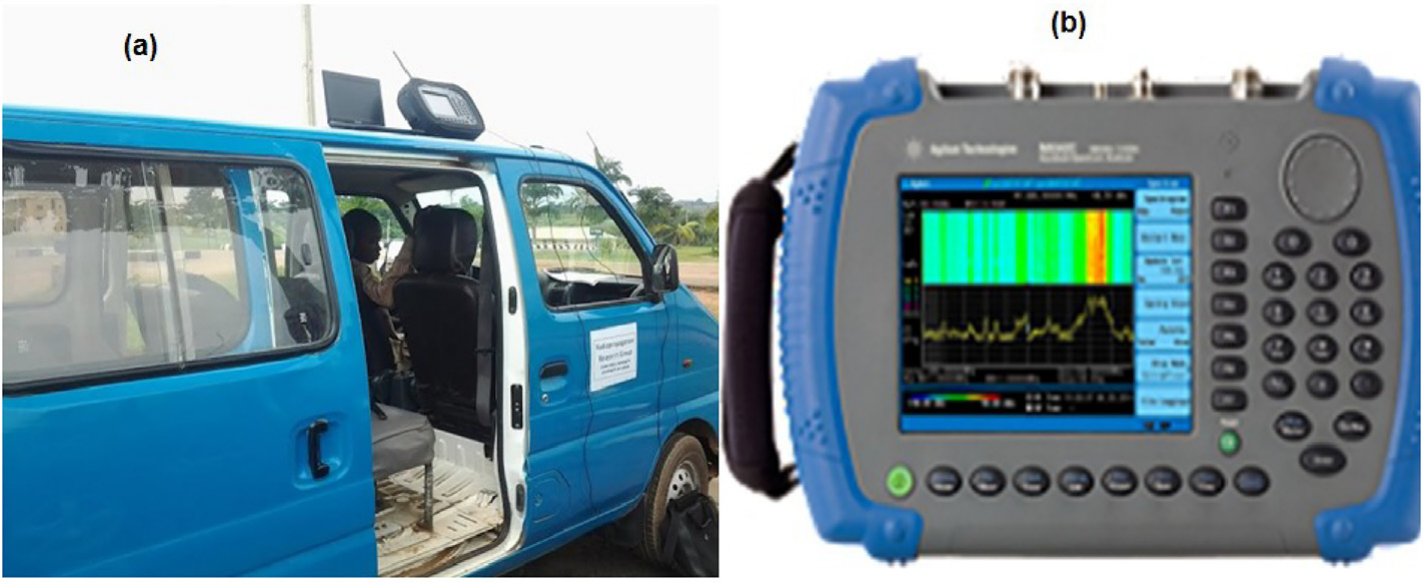
Measurement equipment (a) measurement bus (b) agilent N9342C agilent spectrum analyzer.

Agilent spectrum analyser model N9342C was used as the receiver for the measurements of the electromagnetic field data and the setup is as shown in Figure 1 while, Figure 2(a) and Figure 2(b) show the measurements equipment used which consist of the Van and the spectrum analyser. Table 2 provides details of the configuration parameters used on the spectrum analyser. The analyser has an in-built Global Positioning System (GPS) receiver. For efficient tracking of the satellite receivers, an external GPS receiver operating on the L1 band with a center frequency of 1575.42 MHz was properly fixed on the vehicle roof top. A whip retractable Diamond RH799 omni-directional antenna with frequency range 70 MHz–1000 MHz was coupled on the analyser to capture the signal emitted from the transmitters at predefined distances, usually, far field distances. This receiver was used because the operating frequencies of the all the transmitters considered in these measurements fall within the operating band of the antenna. It was also ensured that the GPS antenna was protected against electromagnetic interference and scattered rays from the whip antenna radiation pattern by employing spatial separation distance between the two antennas. Moreover, the second and third harmonics of the transmitters were all outside the GPS's operating frequency. Therefore, mutual interference between the whip and GPS antennas is not expected. In order to minimize costs and time constraints, taking stand-alone path loss measurements for each transmitter, the channel scanner tool embedded on the analyser was activated and configured with the operating frequencies of the transmitters. This tool created a multi-frequency site survey, which enables scanning of up to 20 channels at once and simultaneously. It further helps to identify potential interference from other sources. By configuring the center frequency of each of the transmitter, an appropriate resolution bandwidth (RBW) and preamplifier were chosen. The electromagnetic field strength of each of the transmitter were logged and saved along-side with the operating frequency, time stamp, date, altitude and the coordinates of the GPS. For storage purpose, an external hard drive was coupled to the analyser and stored all the recorded path loss data. The vehicle was driven at a speed of 40 km/h while the analyser was placed inside it. This speed is however considered the average speed and it was chosen specifically to minimise the Doppler effects. Using this set-up, the path loss measurements were carried out simultaneously, across all the bands and routes.

**Table 2:** Measurement configuration.

N9342C Agilent Spectrum Analyzer	
Frequency Range Impedance	100 Hz-7 GHz 50 Ω
Resolution Bandwidth (RBW)	10 kHz
Preamplifier	20 dB
Displayed Average Noise Level (DANL)	*-*164 dBm/Hz
Receiver Antenna Type	Diamond RH799
Receiver Antenna Gain	2.51 dBi
Average Receiver Height	1.5 m
Antenna Type GPS antenna frequency	Omni directional L1 band

**Figure 3:**
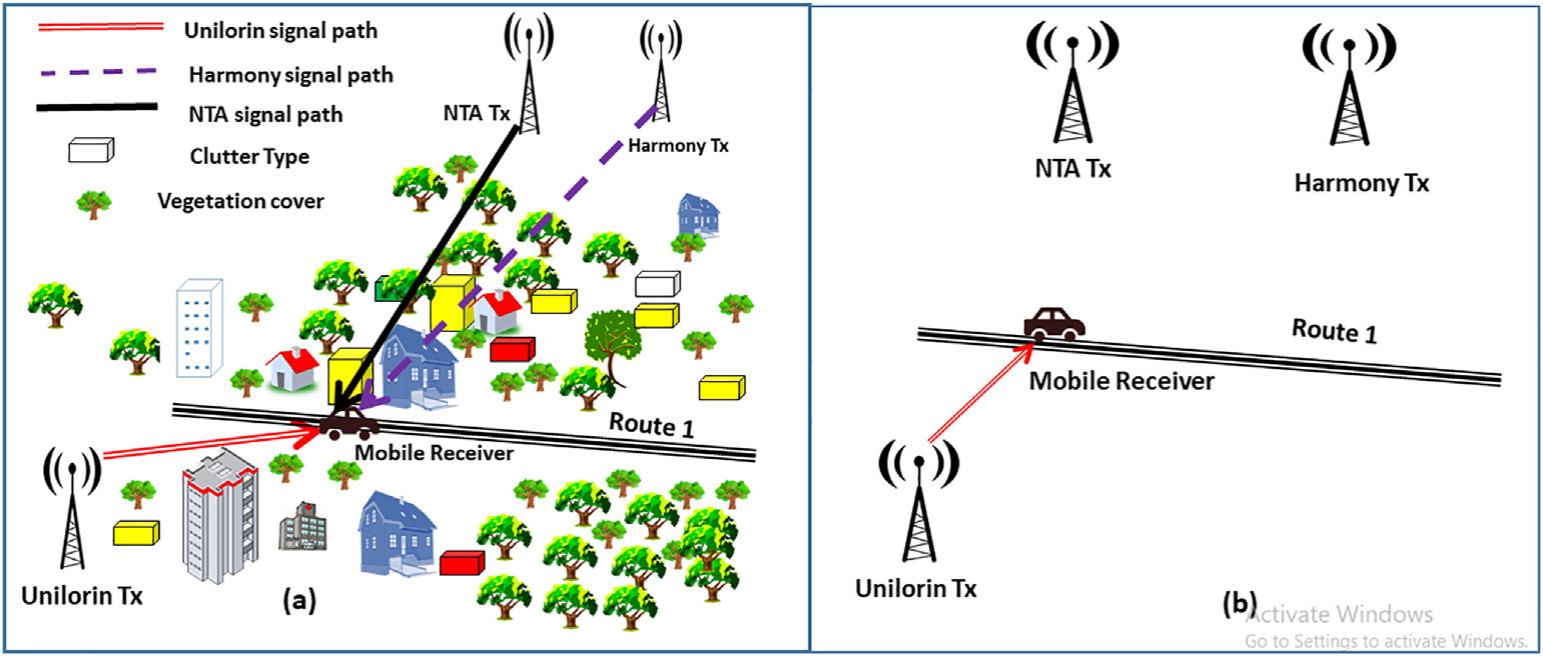
Measurements set-up (a). Proposed multi-transmitter and (b) conventional (standalone).

In both measurements, the transmitters were not co-located and the terrain profile for each measurement route (e.g. route 1 for VHF transmitters) is the same for specific band. But the clutter types along the communication paths are different. This is because as the mobile receiver moves along route 1 for example, the multi-frequency channel scanner enabled on the analyzer records the electromagnetic field strength emanating from the transmitters, alongside the operating frequency, altitude and the coordinates of the GPS. For this, same GPS coordinates and altitudes will be logged. However, the signal paths from the three transmitters are different and so the clutter type. Since each transmitter's communication path with the receiver is distinct and depends on the clutter cover. This new set-up is cost effective and time efficient as few distances will be covered for multiple transmitters. More so, fixed terrain profile and varying clutter types propagations environments are examined as illustrated in Figure 3(a). This is in contrast to the existing and widely used approach for setting-up the instruments for path loss measurements, where, each specific frequency of interest and measurements has to be undertaken along specific routes as illustrated in Figure 3(b). In Figure 3 (b), only Unilorin Transmitter signal strength is measured, therefore, considering X number of transmitters and Y measurements routes to be surveyed, using the existing experimental set-up, measurements have to be repeated (X×Y) times which is usually tedious, expensive and time inefficient. While, the proposed set-up will normalize X to 1 and measurements will be conducted only (1×Y) times since all the transmitters are configured and measurements are taken simultaneously.

### 2.3 Data pre-processing

**Figure 4:**
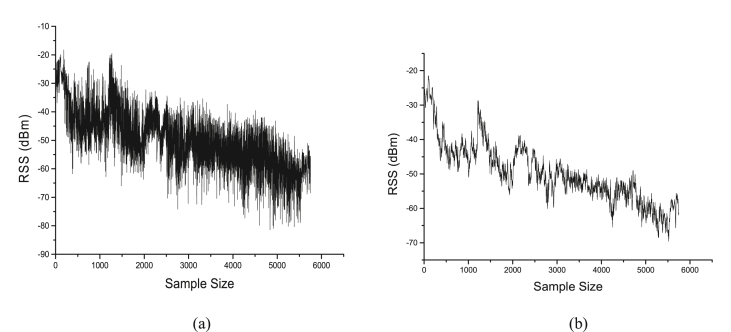
Received signal strength processing (a) before filtering (b) after filtering.

For all the collected electromagnetic field strength measurements data, the small-scale fading characteristics that introduced noise on the signal were removed, resulting to an average received electromagnetic field strength. In other to determine the optimum path loss distance interval that will preserve shadowing effects, the distance of a local mean power was properly chosen. This is to ensure that fast fading is removed, while, shadowing effects are preserved after the averaging process. In Figure 4, the raw received signal strength (RSS) data before and after filtering are shown. Un-weighted sliding average algorithm was used for filtering and smoothing the sample data. This algorithm uses the concept of moving average filter to replace corresponding data points with a mean of the neighborhood data points defined within the span of the measurement. The implementation is provided in Eq. (1) (Chen and Chen, 2003).

(1) $$P\left[n\right]=\frac{1}{M}\overset{M-1}{\underset{i=0}{\sum }}x\left[n-m\right]$$

where;

x[n] is the input sample path loss data, P[n] is the output path loss, M is the filter length and *m* is the sample period. For any given odd values of filter length, M, the output path loss, P[n] is given by:

(2) $$P\left[n\right]=-\frac{1}{M}x\left[n\right]-\ldots -\frac{1}{M}x\left[n-\frac{M-1}{2}\right]+\frac{M-1}{M}x\left[n-\frac{M+1}{2}\right]-\frac{1}{M}x\left[n-\frac{M+3}{2}\right]-\ldots -\frac{1}{M}x\left[n-M+1\right]$$

Eq. (2) is only valid for odd filter lengths. As the filter length increases, the path loss smoothness of the output increases, for this reason, optimum number of candidates’ path loss data points must be chosen to avoid under fitting or over fitting of data. This number has significant effect on the accuracy as smoothing below the optimum value would result to under filtering which will eventually leave noise (small scale fading) on the data. On the other hand, very high number would lead to over fitting which removes the shadowing effects characteristics. In this study, data filtering experiments were conducted for different number of neighboring data points, we implemented the filtering algorithm and tested for various sample points and it was found that fifteen (15) data points gives optimum output. The objective is to choose appropriate sample points that would satisfy the LEE criteria (Lee, 1974, Lee, 1998) for estimating the local mean power values that form the long-term signal along a route. This is also to ensure that after filtering process, the unwanted small-scale fading will be removed and the shadowing (variance) effects would be preserved.

The maximum transmitter and receiver antenna separation distance during this campaign was about 35 km. The far field distance (*d*_*f*_) for each transmitter was computed and the received signal strength emanating from the antenna for separation distances (*d*) of less than 100 m was not analyzed. This is to avoid taking measurements within the near field region. (i.e *d < d*_*f*_). In the far field, the waves propagate and act like plane waves whose power decrease with increase in distance and therefore, the Friis’ free space equation holds in the region beyond the far field distance, (i.e *d > d*_*f*_). Also, it is ensured that all the path loss propagation measurements were conducted in the far field.

### 2.4 Estimation of the measurement distance

Due to nonlinearity of the measurement path, the radial distance between the transmitting and receiving antennas of the propagation measurement routes was considered as it depends on the position of the received signal with respect to the coordinates from the reference point (transmitter's coordinates). This distance is obtained using the distance–coordinate conversion model that gives fifteen significant figures of precision and which uses the spherical law of cosine. With this precision, distances as close to 1 m can be obtained. The model equation is provided in Eq. (3) (Dietert et al, 2020):

(3) $$d\left(km\right)=ACOS\left(\cos\left(a\right)\cos\left(\beta \right)+\sin\left(a\right)\sin\left(\emptyset \right)\right)\times cosR_{E}$$

where, *a* denotes the latitude of the transmitter and β denotes the latitude of the receiver. The longitude of the receiver is represented by ∅,all measured in radians, $R_{E}$ is the radius of the earth in km and the radial distance between the specified transmitter and receiver is denoted by d in km.

## 3 Path loss predictions

### 3.1 Empirical models

The electromagnetic field strength data collected for each transmitter and routes were filtered and converted into path losses. Four empirical path loss propagation models were used to predict the path losses for each scenario, using the transmitters’ system parameters provided in Table 1. The models considered in this work are: the Hata (1980), COST 231 (Erceg, 1999), Egli (1957) and ECC-33 (Abhayawardhana et al., 2005). These models were chosen as they are commonly and widely used empirical models today for prediction of propagation path loss in the VHF and UHF bands under study and they provide benchmark for this study. The propagation parameters used for these models are distance, carrier frequency, transmitter and receiver heights and gains. Details of these parameters are earlier provided in Table 1. Also, correction factors for each scenario were computed. Detailed equations for each of the models used can be found in the references provided.

### 3.2 Kriging Interpolation Method (KIM)

#### 3.2.1 Mesh grid and sampling size estimation

In this work, ordinary Kriging interpolation algorithm was utilized (Krige, 1951, Cressie, 1988). This algorithm uses the concept of regression between the observed neighboring data point to make optimal prediction across the mesh grid (space). The study area was divided into meshes and the positions *(*$x_{i},\;x_{j}$*)* coordinates for each mesh point was computed respectively. The neighborhood of point *‘o’* in the ($X_{o}$) plane was defined and the surveyed points in this neighborhood (sampling) was identified. Assuming u*,* is a point in which the path loss prediction is to be made, and *V* (*u*) = {1 ... *Nu*} are set of points within the surrounding point *u*, with known path loss data, for each point. With neighborhood of point *u*, on a plane *(*$x_{i},\;x_{j}$*)*, the points surveyed in this neighborhood were mapped to path loss data that were sampled considering the total data. For each transmitter and route optimum mesh grid size was obtained by varying the mesh grid from 100 to 500 to obtain the minimum variance.

#### 3.2.2 Estimation of variogram and lag distance

Despite the direction and to make sure that the variance between the measured path losses is of the same distance, the isotropic random fields are considered. Positive values of lag distance $\left(h_{u}\right),\;u=1,\ldots U$ is presented. With this kind of arrangement, the lag distances are assumed in such way that $h_{u}<h_{u+1}$ are reported as absolute separation from the point of origin. The lag distances, $N\left(h_{u}\right)$ and the experimental variogram ${\hat{\gamma }}^{M}\left(h_{u}\right)$were computed for a range of lags using Eqs. (4) and (5), respectively (Abdulrasheed et al., 2017).

(4) $$N\left(h_{u}\right)=\left\{\left(X_{i}-X_{j}\right)∶h_{u}-\frac{\delta_{u}}{2}\leq ||X_{i}-X_{j}||<h_{u}+\frac{\delta_{u}}{2}\delta_{u}\right\}$$

(5) $${\hat{\gamma }}^{M}\left(h_{u}\right)=\frac{1}{2\left|N\left(h_{u}\right)\right|}\overset{N\left(h_{u}\right)}{\underset{1}{\sum }}{\left[Z\left(X_{i}\right)-Z\left(X_{j}\right)\right]}^{2}$$

where, $Z\left(X_{i}\right)$, $X_{i}$, and $X_{j\;}$ are the measured path loss, sampled location and neighbouring location at a lag haway. Here, $X_{i}\in R^{d},\;i=1,\ldots N$ are the points locations of the data. The lag distance $N\left(h_{u}\right)$, consists of path loss measured points, whose shared separation are in the range $\left[\left(h_{u}-\frac{\delta_{u}}{2},h_{u}+\frac{\delta_{u}}{2}\right).N\left(h_{u}\right)\right]=\left|N\left(h_{u}\right)\right|$ will denote cardinality of class $N\left(h_{u}\right)$. The semivariogram was fitted with the spherical model using Eq. (6).

(6) $$\gamma_{\theta }\left(h\right)=\left\{ \begin{array}{ll} 0\;, & h=0\\ c_{o}+c_{1}\left(\frac{3}{2}\left(\frac{\left|h\right|}{c_{2}}\right)-\frac{1}{2}{\left(\frac{\left|h\right|}{c_{2}}\right)}^{3}\right), & 0<h\leq c_{2}\\ c_{o}+c_{1}\;, & h>c_{2} \end{array}\right.$$

With $\left(c_{o},c_{1},c_{2},\right),c_{i}\geq 0$ for i=0,1,2.

Where, θ, $c_{o}$, $c_{1}$ and $c_{2}$donates the parameters of free vector, nugget effect, sill and range respectively. The free vector parameters decide the shape of variogram. The nugget effect limit is nonzero $\lim_{h\to 0}\gamma \left(h\right)=c_{o\;}$and sill limit is set to be $\lim_{h\to +\infty }\gamma \left(h\right)=+\infty$.

#### 3.2.3 Kriging variance estimation

The predicted path loss was calculated as a linear combination of the weight ($W_{i}$) and the neighborhood (known) path loss ($Z_{i}$) using Eq. (7). For error variance to be minimized by the set of weights, a constraint is introduced in Eq. (8). By this and under unbiased conditions, the mean error is zero.

(7) $$\hat{\;Z}\left(X_{o}\right)=\overset{N}{\underset{i=1}{\sum }}W_{i}Z\left(X_{i}\right)$$

where

(8) $$\begin{array}{l} \sum_{\begin{array}{l} i=1 \end{array}}^{N_{-}}W_{i}=1 \end{array}$$

The Kriging weights are derived as in Eq. (9):

(9) $$\underset{\left(N+1\right)\times 1}{\underset{︸}{\left[\begin{array}{l} W_{1}\\ W_{2}\\ \vdots \\ W_{N}\\ \lambda \end{array}\right]}}={\underset{\left(N+1\right)\times \left(N+1\right)}{\underset{︸}{\left[\begin{array}{lllll} \gamma \left(X_{1},X_{1}\right) & \gamma \left(X_{1},X_{2}\right) & \cdots & \gamma \left(X_{1},X_{N}\right) & 1\\ \gamma \left(X_{2},X_{1}\right) & \gamma \left(X_{2},X_{2}\right) & \cdots & \gamma \left(X_{2},X_{N}\right) & 1\\ \cdots & \cdots & \cdots & \cdots & 1\\ \gamma \left(X_{N},X_{1}\right) & \gamma \left(X_{N},X_{2}\right) & \cdots & \gamma \left(X_{N},X_{N}\right) & 1\\ 1 & 1 & 1 & 1 & 0 \end{array}\right]}}}^{-1}\cdot \underset{\left(N+1\right)\times 1}{\underset{︸}{\left[\begin{array}{l} \gamma \left(X_{0},X_{1}\right)\\ \gamma \left(X_{0},X_{2}\right)\\ \vdots \\ \gamma \left(X_{0},X_{N}\right)\\ 1 \end{array}\right]}}$$

Using the matrix of variograms, Γ and vector of variogram and $\gamma_{o}=\gamma \left(X_{o},X_{i}\right)$, then, the set of weights, $W_{o\;}$ is derived as:

(10) $$W_{o}\;=\Gamma^{-1}\cdot \gamma_{o}$$

where $\Gamma_{i,j}=\gamma \left(X_{i},X_{j}\right)$ and $\;\gamma_{o}=\gamma \left(X_{o},X_{i}\right)$. Therefore, from (10), the Kriging Variance, ($\sigma_{ok}^{2})$ is calculated using Eq. (11):

(11) $$\sigma_{ok}^{2}=\lambda +\overset{N}{\underset{i=1}{\sum }}W_{i}\gamma \left(X_{o},X_{i}\right)$$

The Lagrange parameter is represented byλ, and it was introduced to reduce the Kriging error.

## 4 Evaluation metrics

Performance metrics are used to evaluate the accuracy and efficiency of the predictive models relative to the measured path loss. The metrics are: Mean Prediction Error (MPE) which gives the biasness of the predictive model; Root-Mean-Squared Error (RMSE) which indicates the variance in the errors. For urban deployments, values within the range 0–6 dB are acceptable while, in rural and suburban area, values up to 10 dB are still acceptable (Faruk et al., 2013a); Standard Deviation Error (SDE), which indicates the degree of deviation from the mean; Spread Corrected Root Mean Square Error (SC-RMSE), similar to RMSE but useful for noisy links; Efficiency (EF) which was introduced in (Greenwood et al., 1985, Vicente-Serrano et al., 2003); Error Rate and Gaussian Kernel Density Estimation (GKDE).

### 4.1 Mean Prediction Error (MPE)

The Mean Prediction Error (MPE) can be used to give an indication of the bias of predictions, i.e. it determines if the model is more likely to under-predict or over-predict. The MPE is calculated using Eq.(12), which is the average of the difference between the measured path loss and the model's predictions. The value of MPE close to zero indicates better fitness. It is measured in dB.

(12) $$MPE=\frac{1}{n}\overset{n}{\underset{i=1}{\sum }}\left(Z_{P,i}-Z_{m,i}\right)$$

where n is the number of samples, $Z_{Pi}$ and $Z_{m,i}$ are the model's predicted and measured path loss at a given point irespectively. Using similar notation.

### 4.2 Root-Mean-Squared Error (RMSE)

The RMSE values give an indication of the variance in the errors. Since the larger errors are given more weight by squaring the errors in the RMSE approach. It is expressed using Eq. (13). The RMSE of 0–6 dB are acceptable in urban deployment, while 0–10 dB for rural and suburban area.

(13) $$RMSE=\sqrt{\frac{1}{n}\overset{n}{\underset{i=1}{\sum }}{\left(Z_{P,i}-Z_{m,i}\right)}^{2}}$$

### 4.3 Standard Deviation Error (SDE)

The Standard Deviation Error (SDE) is a measure of the degree of the deviation of the errors from the average value and it is expressed in Eq. (14).

(14) $$SDE=\sigma =\sqrt{\frac{1}{n}\overset{n}{\underset{i=1}{\sum }}{\left(Z_{p,i}-{\bar{Z}}_{p}\right)}^{2}}$$

where, $\;{\bar{Z}}_{p}$ is the mean of the predicted path loss.

### 4.4 Spread Corrected Root Mean Square Error

Spread Corrected Root Mean Square Error (SC-RMSE) is the absolute value of errors reduced by the SDE of the measurements and it is expressed in Eq. (15).

(15) $$SCRMSE=\sqrt{\frac{1}{n}\overset{n}{\underset{i=1}{\sum }}{\left(\left|Z_{P,i}-Z_{m,i}\right|\right)}^{2}-\sigma }$$

### 4.5 Efficiency (EF)

A similar method referred to as the model efficiency (EF) is proposed by the authors in (Greenwood et al., 1985). The EF is determined using Eq. (16).

(16) $$EF=1-\frac{\sum_{i=1}^{n}{\left(Z_{p,i}-Z_{m,i}\right)}^{2}}{\sum_{i=1}^{n}{\left({\bar{Z}}_{m}+Z_{m,i}\right)}^{2}}$$

where ${\bar{Z}}_{m}$ denotes the mean of the measured path loss.

The value of EF is desired to be as close to one as possible. In cases where EF is very close to zero, the authors of Vicente-Serrano et al. (2003) found that the mean of the observations gives more reliable estimations than the model.

### 4.6 Error rate

The percentage error gives you the difference between the approximate and exact value as a percentage of the exact value. Mathematically expressed as:

(17) $$Error\;rate=\frac{\left|Z_{P,i}-Z_{m,i}\right|}{Z_{P,i}}\times 100$$

### 4.7 Gaussian Kernel Density Estimation (GKDE)

The Gaussian KDE is a measure of the skewness of the mean of a given set of data towards zero. It is also used to estimate the probability distribution function of a given data and it is expressed as follows:

(18) $$G_{D}\left(y_{i}:\beta \right)=\frac{1}{\sqrt{2\pi }\beta }e^{-\frac{{y_{i}}^{2}}{2\beta^{2}}}$$

where $y_{i}$ is the given data set which is the set of prediction errors for this research and β is the width or boundary of the prediction errors.

## 5 Results and discussion

**Figure 5:**
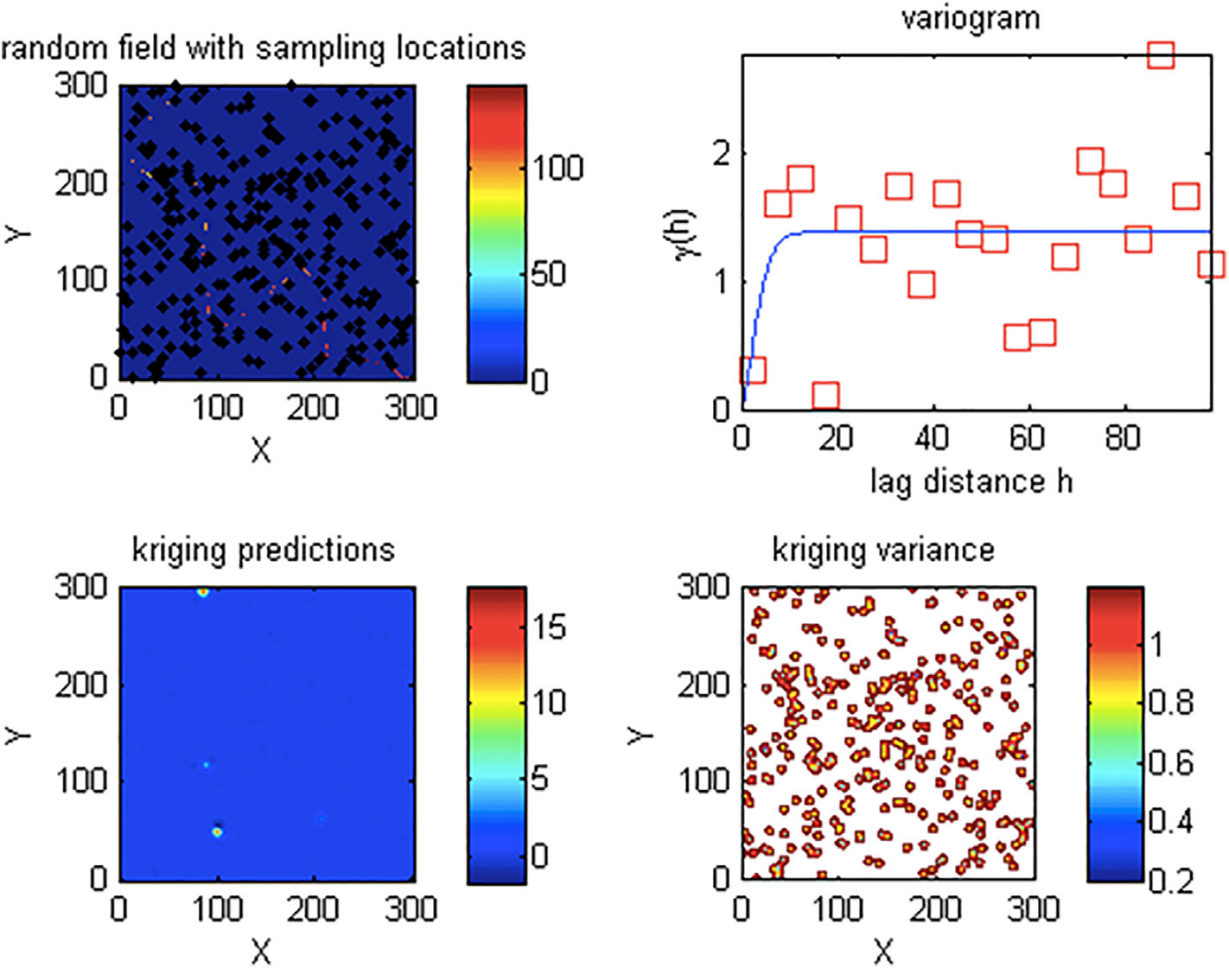
Unilorin FM transmitter Kriging Maps for route 1 (a) sampling locations (b) variogram (c) Kriging predictions and (d) kriging variances.

The kriging algorithm and empirical path loss models were implemented. This is used to visualize the KIM maps showing the sampling locations of the random field for all the transmitters across all the measurement routes. For each random field, a specified sample size was used; in order to ensure minimal variance and to achieve optimality in prediction. As an illustration, we show only the results for only Unilorin transmitter along route 3. In Figure 5 (a), the sampling locations for a size of 300 is presented. The spherical variogram with its corresponding distance which is lag is given in Figure 5 (b). Figure 5 (c) presents the Kriging predictions while, the Kriging variance is presented in Figure 5 (d).The kriging variances are depicted with a contour to illustrate different values.

**Figure 6:**
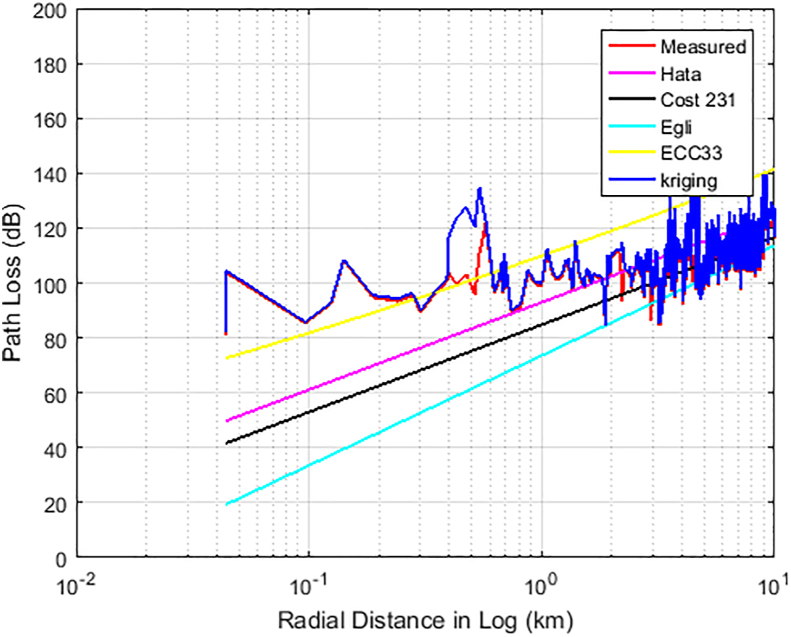
Path Loss Measured and Predicted for 89.3 MHz along route 1.

**Figure 7:**
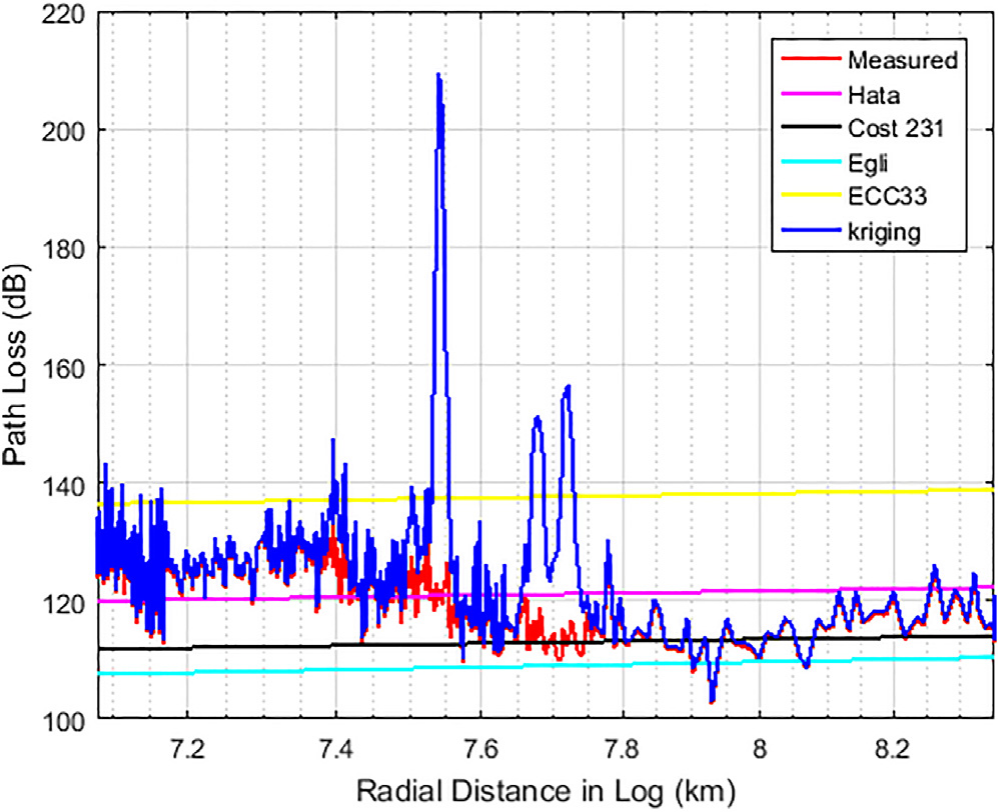
Path Loss Measured and Predicted for 89.3 MHz along route 2.

**Figure 8:**
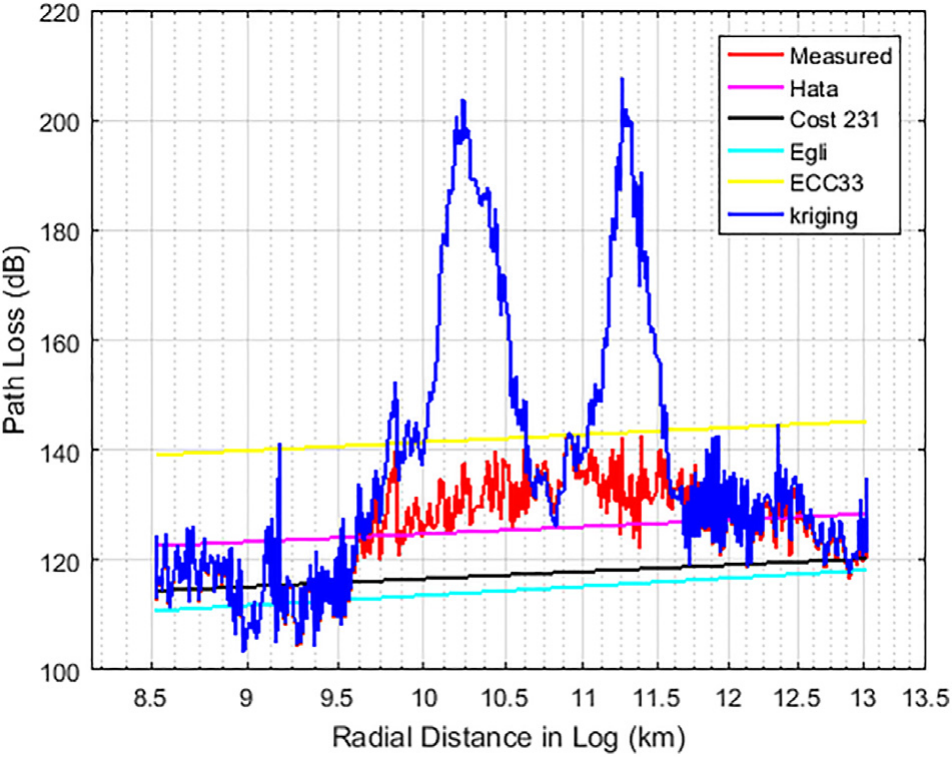
Path Loss Measured and Predicted for 89.3 MHz along route 3.

Figures 6, 7, and 8 show the comparison between the actual measured path losses and the predicted path losses using the Kriging and empirical path loss models for Unilorin transmitter with operating frequency of 89.3 MHz along routes 1–3. In Figure 6, large scale fading and shadowing were noticeable as the loss increases with increase in distance. On this route, the losses are predominately large scale due to reflection, and diffraction of the signal from buildings, and multipath effects along the route. This route is within the University campus and is categorised as an urban settlement. The Min-Max loss of 84 dB–140 dB was measured with an average loss of 111.8 dB. Hata model is one of the empirical models whose predictions were found to be of good fit for path losses that are measured at *d >* 2 km, so also is COST 231. On the other hand, the Egli and ECC-33 models, deviate significantly from the mean measured path loss because of the under-prediction observed for Egli while over prediction was observed for ECC-33. In the case of KIM, predictions were quite optimum as they followed the measured path loss, with some spikes due to interspace distance between the sample points. However, the situation is found different along route 2 as the communication path between the transmitter (UNILORIN) and the receiver was less than 2 km and the clutter types were different. The buildings in the area are densely distributed and the average loss measured along this route was 120 dB. This is about 8 dB higher than that of route 1. Terrain irregularities was found to be the major contributing factor, as the measured loss does not exhibited ideal situation as shown in Figure 7. In Figure 8, how the loss varies with distance along the communication path is shown. This area is quite a busy road and the average loss was 125 dB. None of the models predicted the loss, even though Hata model's prediction was found to be optimum among the contending models. Also, along this route, large spikes were observed for the Kriging model.

**Figure 9:**
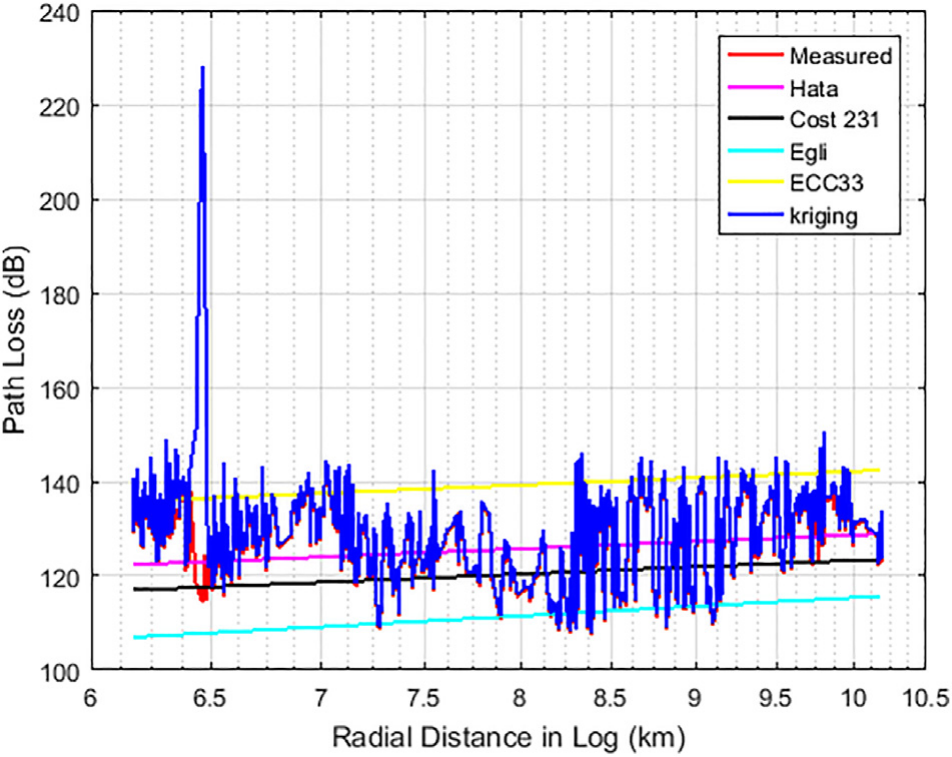
Path Losses Measured and Predicted for 203.25 MHz along route 1.

**Figure 10:**
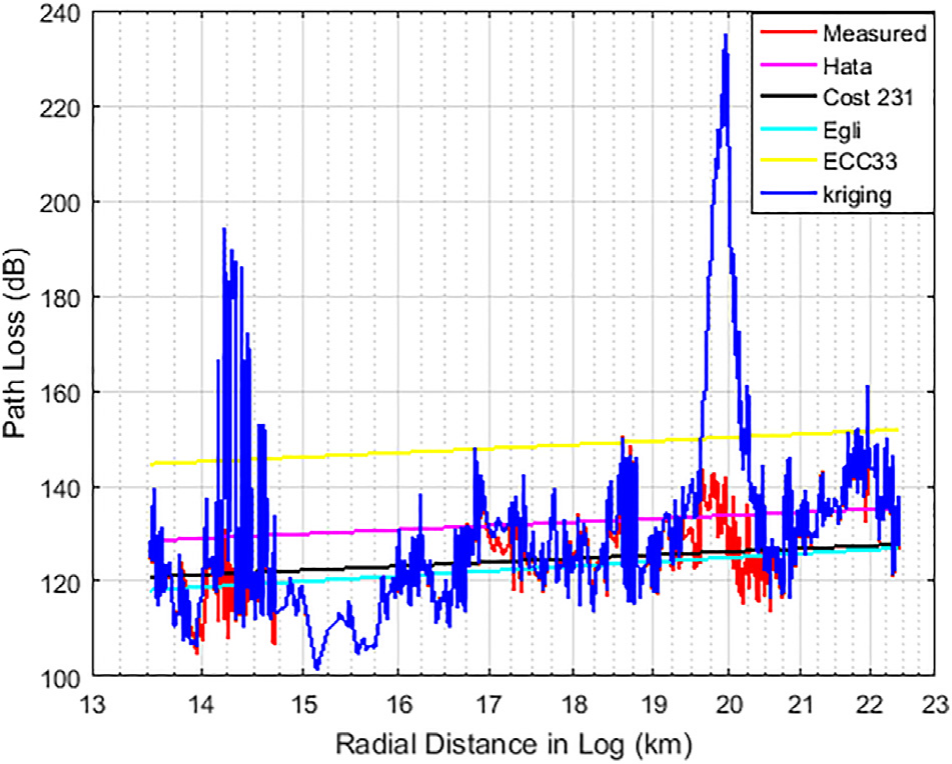
Path Losses Measured and Predicted for 103.50 MHz along route 1.

Figures 9 and 10 show how the measured path loss varies with distance for NTA Ilorin and Harmony transmitters. Due to page budget, we could not show for other transmitters (i.e. NTA osogbo, NDTV IBOKUN, OSBC OSOGBO, NTA ILE-IFE) and routes (routes 2b–3b). But statistical analyses for each of the model relative to the measured loss are provided in Tables 3–9.

**Figure 11:**
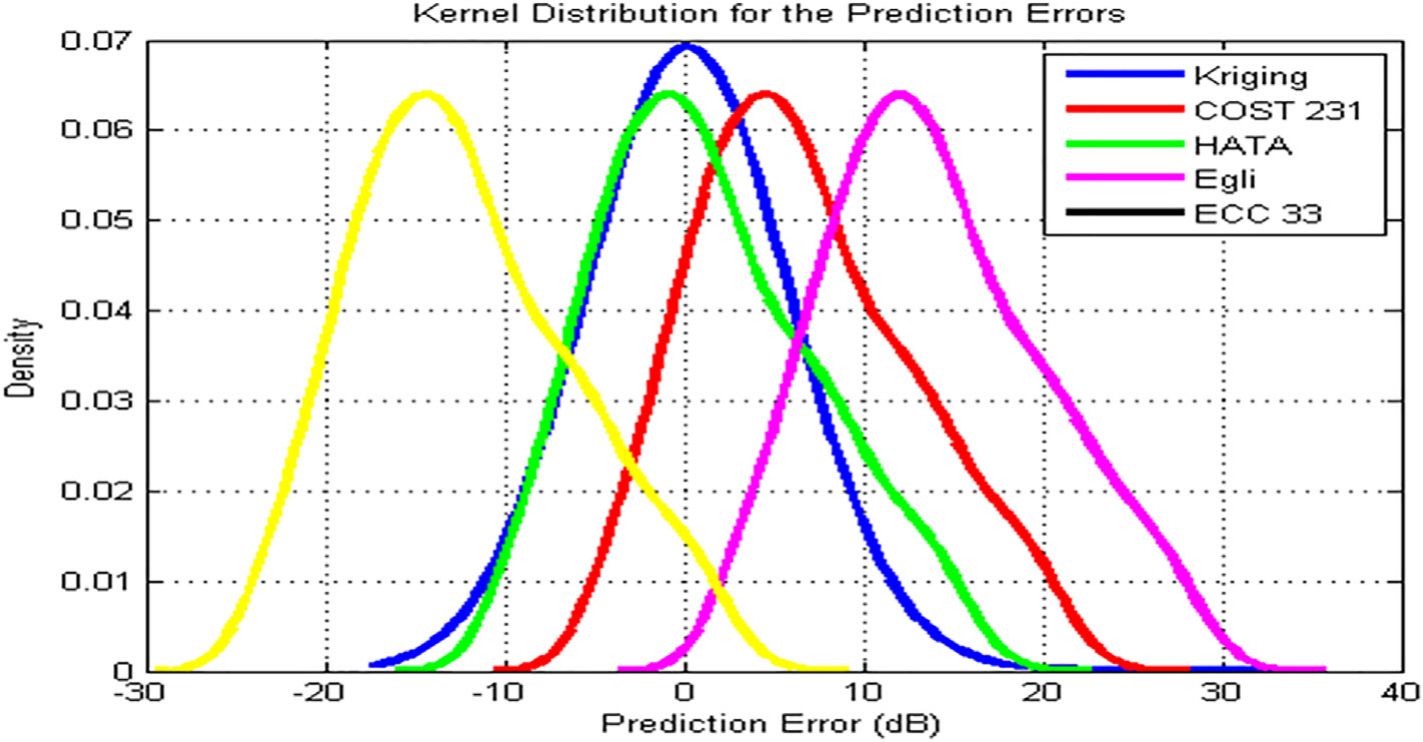
NTA Ilorin Transmitter model Kernel distribution of the prediction errors along route 2.

**Figure 12:**
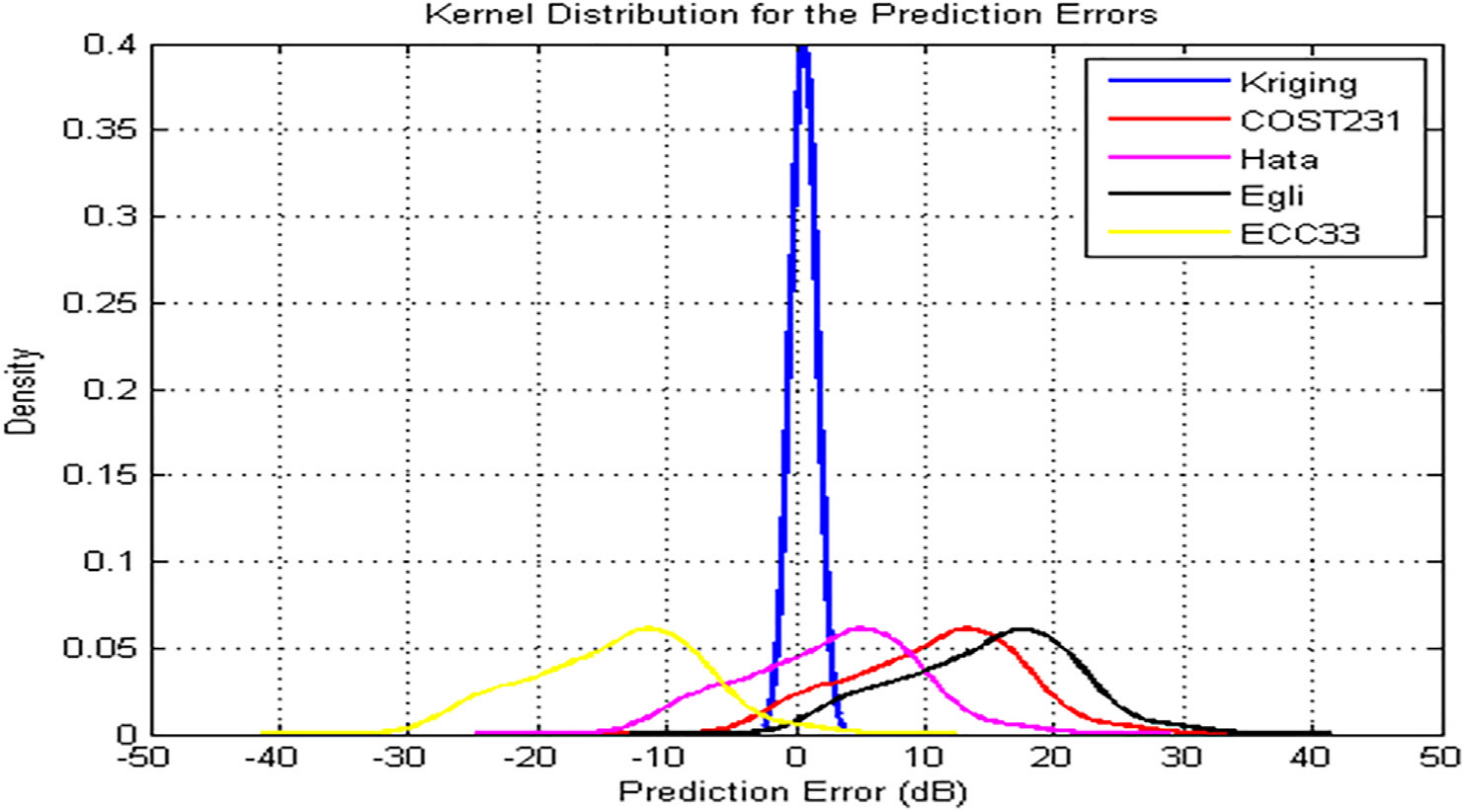
Harmony FM Transmitter model Kernel distribution of the prediction errors along route 3.

Figures 11 and 12 depict the kernel density estimation for NTA and Harmony transmitters along routes 2 and 3 respectively. In Figure 11, the kernel density estimation of the prediction error for the kriging method is skewed symmetrically to the mean error of zero. This method achieves the highest density of 0.07. The prediction error of Hata and COST 231 models are also distributed symmetrically, with mean values of -1.2 dB and 4.0 dB respectively. The prediction error of the Egli and the ECC-33 models also followed the Gaussian normal distribution but deviated significantly, from true density. The ECC-33 model negatively skewed, while Egli model positively skewed. The situation is found different in Figure 12 for Harmony transmitter where both Hata and COST 231 models were largely, positively skewed. The Kriging, Egli and ECC-33 models maintained same performance. It is important to note that except Kriging, all other models’ prediction error do not follow normal Gaussian distribution. However, considering route-on-routes specific performance, it can conclude that Gaussian normal distribution can perfectly characterize the measured losses and so the prediction errors of the models relative to the measured losses.

**Figure 13:**
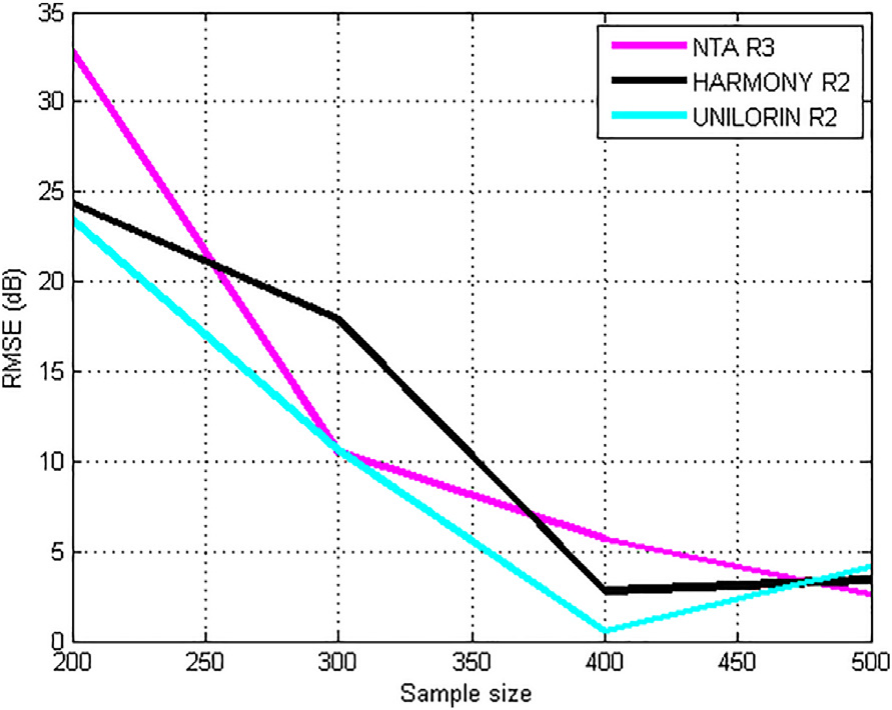
RMSE and Sample Size for KIM along route 2 for VHF transmitters.

**Figure 14:**
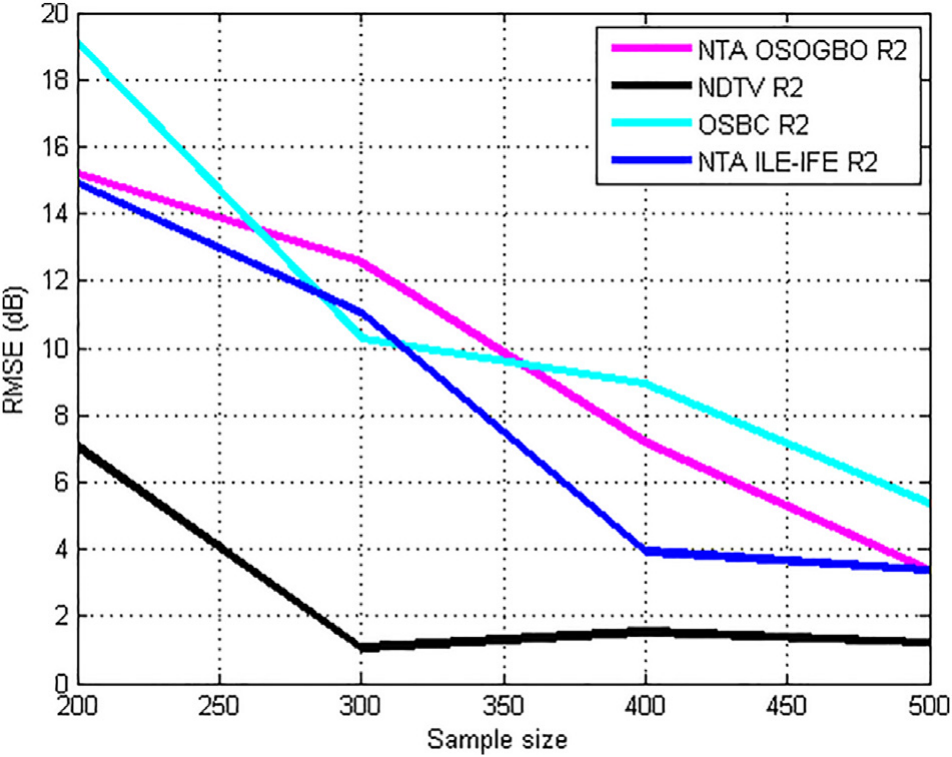
RMSE and Sample Size for KIM along route 2 for UHF transmitters.

Figures 13 and 14, show the effect of sample size on the prediction accuracy of kriging method. The study area was divided into meshes and the positions *(*$x_{i},\;x_{j}$*)* coordinates for each mesh point was computed respectively. The neighborhood of point *‘o’* in the ($X_{o}$) plane was defined and the surveyed points in this neighborhood (sampling) was identified. For each transmitter and routes, optimum mesh grid size was obtained by varying the mesh grid from 100 to 500 to obtain the minimum variance. From the results, it have been observed varying prediction accuracies for different sample sizes. The method achieves optimality at specific sample size and thereafter, the error began to increase. In Figure 13, the RMSE at different sample size for the three transmitters are presented. The KIM has RMSE of 2.59 dB and 1.73 dB for Harmony and Unilorin transmitters at sample size was 400 which are the least values. On the other hand, NTA reaches optimized prediction with RMSE value of 2.59 dB at a sample size of 500, this is very suitable for urban area. Figure 14 shows RMSE for different sample sizes on KIM for the four distinct UHF transmitters. The optimal sample size as in the case of NDTV transmitter was 300, while for others, the sample size had to be increased to 500 to achieve optimality. This is an indication that for optimality to be achieved, the sample size must be appropriately chosen when deploying Kriging method for path loss prediction.

**Figure 15:**
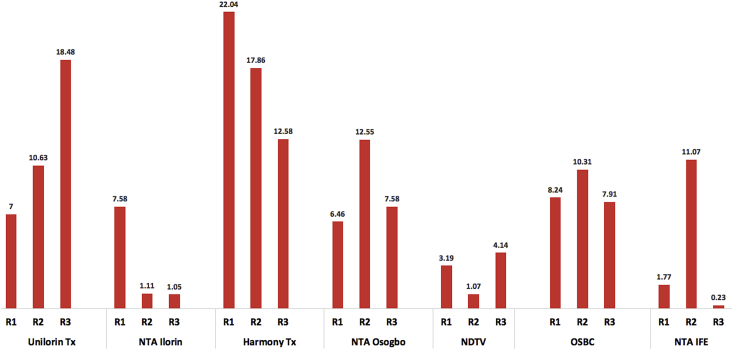
Validation of kriging model.

In Figure 15, the validation results for the Distance-Based Kriging path loss propagation model is presented. The model was validated across the multi-bands and devise set of environments characterized with different routes. Typically, the Root-Mean-Squared Error (RMSE) metric is used to test the validity and gauge the performance of terrestrial radio propagation models. The RMSE indicates the variance in the errors, values in between 0-6 dB are acceptable, for models developed for urban environments, sometimes up to 10 dB marginal error, could still be acceptable for rural and suburban deployments (Faruk et al., 2013). The Figure provides route specific performance of the model. Based on the established metrics, the KIM performs best for the NDTV (479.25 MHz) as the route-on-route RMSE were all below the benchmark of 6 dB. Similarly, the model's performance was excellent for NTA Ilorin (203.25 MHz) with an average RMSE value of 6.40 dB. The model performed awfully for the Harmony Tx (103.5 MHz) and Unilorin Tx (89.3 MHz) with average RMSE values of 17.0 dB and 12.04 dB respectively. These are higher than the benchmark. The average RMSE for OSBC transmitter (559.25 MHz), NTA, IFE (615.25 MHz) and NTA Osogbo (695.25 MHz) are 7.43 dB, 5.41 dB and 8.08 dB respectively. These values are within the acceptable error margin for channel model deployment in the prediction of path losses for wireless systems.

Tables 3, 4, 5, 6, 7, 8 and 9 provides detailed statistical analysis in terms of mean error, root mean square error, spread corrected mean square error and staon error for each model relative to the measured propagation losses. The analysis is provided for each method used and across all the measurement routes. All the transmitters were considered for this exercise. Detailed statistical results for Unilorin transmitter is shown in Table 3. The Hata model has the lowest RMSE, while, ECC-33 has the highest. The route-on-route average RMSE values of the models are: 9.11 dB, 12.04 dB, 12.48 dB, 16.5 dB and 18.62 dB respectively for Hata, Kriging, COST 231, Egli and ECC-33 models. In terms of the mean prediction error, COST 231, Hata and Egli under-estimate the path loss while, ECC-33 and Kriging over-estimate the loss. The situation was found to be similar for NTA Ilorin and Harmony transmitters as shown in Tables 4 and 5 respectively, except in Table 5 where Hata over-estimate the loss with a positive mean average.

The Egli and ECC-33 consistently maintained similar performance relative to the measured path loss. Noting the fact that RMSE and MPE are the most widely used metrics to gauge model performance. Critical evaluation shows that the KIM method yielded good results across the routes and bands. However, the standard deviation error for this method was found to be on the high side as method uses the concept of regression between the observed neighboring data point to make optimal prediction across the mesh grid (space). This resulted to a high SDE far above the empirical path loss propagation models. In Table 3, the average SDEs for the empirical models are all below 7.0 dB, while about 15 dB was recorded for the KIM method. These high deviations were as a result of the spikes observed since the prediction could only be possible based on the measured path loss. On the other hand, the empirical models’ predictions are independent on the measurement samples. For this reasons, despite the high mean prediction and root mean square errors recorded for the Egli and and ECC-33 models, the standard deviation errors were found to be low. As expected, the measured mean standard deviation errors for the UHF frequencies are found to be higher than that of VHF frequencies. This is because the UHF frequencies have shorter wavelength and therefore, reflection, diffraction and signal absorption are more significant.

## 6 Conclusion

This paper introduced a cost effective and time efficient set-up for large scale multi-transmitter path loss propagation measurements in the VHF and UHF bands. The set-up was found efficient in examining both the effect of clutter and terrain. The measurement can be conducted simultaneously across many frequencies. The methodology employed preserves the shadowing effects on the measured path loss data and removes the small-scale fading characteristics that introduced noise on the signal. An approach for determining the optimum path loss distance interval that will preserve shadowing effects was provided. The paper demonstrates how filtering algorithm could be used to remove noise part of the data.

Furthermore, the measured path loss data were used to provide the practical error bound of some predictive path loss propagation models. The models considered were grouped into empirical and geospatial. Among the empirical models, the widely used/deployed models (Hata, COST 23, Egli and ECC-33) were considered, while, only kriging was chosen amongst the geospatial methods.

Findings revealed that large scale fading and shadowing were noticeable as the loss increases with increase distance. The measured losses were predominately large scale due to reflection, and diffraction of the signal from buildings, and multipath effects along the routes. Findings also show that as the filter length increases, the path loss smoothness of the output increases, for this reason, optimum number of candidates’ path loss data points must be chosen to avoid under fitting or over fitting of data.

The empirical models’ predictions were optimum relative to the measured path losses with Hata and COST 231 models providing good fitness with respect to path loses measured. The Egli and ECC-33 models, deviate significantly from the mean measured path loss as both models, mainly, and consistently, under-predicted or over-predicted the path loss across the frequency bands and measurement routes. On the other hand, KIM predictions were quite optimum as they followed the measured path loss, with some spikes due to interspace distance between the sample points. The Standard deviation errors for all the empirical models were however low when compared to the Kriging.

Considering route specific performance, Gaussian normal distribution can perfectly characterize the measured losses. The kernel density estimates of the prediction errors for the Kriging method is skewed symmetrically to the mean error of zero. The prediction error of Hata and COST 231 models are also distributed symmetrically, with mean values of -1.2 dB and 4.0 dB respectively. The prediction error of the Egli and the ECC-33 models also followed the Gaussian normal distribution but deviated significantly, from true density. The ECC-33 model negatively skewed, while Egli model positively skewed.

Furthermore, the Kriging method has varying prediction accuracies for different sample sizes. The method achieves optimality at specific sample size. However, prediction outside the optimal grid size, yielded high prediction error, beyond that of the empirical models. It was also found that optimized and accurate prediction could be achieved with suitable sample size. It was also found that the KIM method minimizes the cost of measurements, analysis and predictions of path loss in built-up propagation environments. Since predictions can be achieved with few sample size.

In future work, it is hoped that the sudden overshoot in path loss prediction of the KIM at some distances from the transmitters would be investigated. It is also worth investigating the effects of other fitted variogram models in KIM predictions.

## Declarations

### Author contribution statement

Nasir Faruk & Aderemi A. Atayero: Conceived and designed the experiments; Performed the experiments; Wrote the paper.

I. Y. Abdulrasheed, Abubakar Abdulkarim & Olugbenga Sowande: Performed the experiments; Analyzed and interpreted the data.

N. T. Surajudeen-Bakinde: Analyzed and interpreted the data; Wrote the paper.

Emmanuel ADETIBA & Ayodele H. IFIJEH: Contributed reagents, materials, analysis tools or data; Wrote the paper.

A. A. Oloyede: Conceived and designed the experiments; Performed the experiments.

### Funding statement

This work was supported by the Communication and Network Research group (CNRG), University of Ilorin, Nigeria and 10.13039/501100012497Covenant University Centre for Research, Innovation and Development (CUCRID), Nigeria; IoT-enabled Smart and Connected Communities (SmartCU) Research Cluster of Covenant University, Nigeria

### Data availability statement

Data will be made available on request.

### Declaration of interests statement

The authors declare no conflict of interest.

### Additional information

No additional information is available for this paper.

## Figures and Tables

**Table 3:** Error analysis for the models of unilorin FM transmitter.

MODEL		ROUTE 1	ROUTE 2	ROUTE 3	AVERAGE
KRIGING	RMSE (dB)	7.0028	10.6392	18.4815	12.0412
	SC-RMSE (dB)	11.8906	12.7863	21.4611	15.3793
	ME (dB)	1.92E+00	3.28E+00	7.51E+00	4.24E+00
	SD (dB)	11.7945	11.129	21.104	14.6758
	Error Rate (%)	1.7648	2.6651	5.9671	3.4657
COST 231	RMSE (dB)	13.3914	12.5391	11.5141	12.4815
	SC-RMSE (dB)	12.3974	12.059	10.1023	11.5196
	ME (dB)	-5.6607	-10.654	-8.4042	-8.2396
	SDE (dB)	16.095	0.5542	1.7302	6.1265
HATA	RMSE (dB)	12.389	7.0703	7.8746	9.1113
	SC-RMSE (dB)	10.9216	6.6148	6.5251	8.0205
	ME (dB)	2.4897	-2.5036	-0.2538	-0.0892
	SDE (dB)	16.095	0.5542	1.7302	6.1265
EGLI	RMSE (dB)	19.7392	16.2172	13.5809	16.5124
	SC-RMSE (dB)	16.6531	15.5834	11.7536	14.6634
	ME (dB)	-12.1005	-14.7672	-11.2064	-12.6914
	SDE (dB)	20.2452	0.6971	2.1763	7.7062
ECC-33	RMSE (dB)	22.2379	15.4499	18.1985	18.6288
	SC-RMSE (dB)	9.7745	14.9309	16.5764	13.7606
	ME (dB)	19.2364	13.9577	16.427	16.5404
	SDE (dB)	14.9815	0.5733	1.8168	5.7905

**Table 4:** Error analysis for transmitter with 203.25 MHz.

MODELS	METRICS	Route 1	Route 2	Route 3	Average
KRIGING	RMSE (dB)	7.58	1.11	1.05	6.40
	SC-RMSE (dB)	11.8224	5.8502	12.5543	10.0756
	MPE (dB)	1.4334	0.2532	3.3583	1.6816
	SDE (dB)	10.635	6.0057	11.1179	9.2529
	Efficiency	0.9991	1	0.9983	0.9991
	Error Rate (%)	1.1156	0.1926	2.6307	1.313
COST 231	RMSE (dB)	13.144	9.5645	7.4858	10.0648
	SC-RMSE (dB)	11.381	8.9429	6.8723	9.0645
	ME (dB)	-9.8362	-7.3712	-1.5886	-6.2653
	SDE (dB)	2.0759	0.7827	0.7456	1.2014
HATA	RMSE (dB)	9.789	6.4099	8.2421	8.147
	SC-RMSE (dB)	8.1056	5.8138	7.6489	7.1894
	ME (dB)	-4.4504	-1.9854	3.7973	-0.8795
	SDE (dB)	2.0759	0.7827	0.7456	1.2014
EGLI	RMSE (dB)	21.2107	16.2176	11.2419	16.2234
	SC-RMSE (dB)	18.723	15.2583	10.442	14.8078
	ME (dB)	-19.2574	-15.011	-8.5644	-14.2776
	SDE (dB)	2.7633	1.0419	0.9925	1.5992
ECC-33	RMSE (dB)	12.53	13.0052	18.768	14.7677
	SC-RMSE (dB)	10.9527	12.3052	18.0789	13.779
	ME (dB)	8.9976	11.4883	17.284	12.59
	SDE (dB)	2.0839	0.7874	0.7508	1.2074

**Table 5:** Error analysis for transmitter with 103.50 MHz.

MODEL		ROUTE 1	ROUTE 2	ROUTE 3	AVERAGE
KRIGING	RMSE (dB)	22.0458	17.8683	12.5866	17.5003
	SC-RMSE (dB)	24.699	18.9919	15.1664	19.6191
	MPE (dB)	8.55	6.88	4.29	6.57
	SDE (dB)	23.0308	17.2663	13.9516	18.0829
	Error rate (%)	6.7662	5.1621	3.1897	5.0393
COST 231	RMSE (dB)	8.8506	9.6828	10.1935	9.5756
	SC-RMSE (dB)	7.3454	9.623	9.9634	8.9773
	ME (dB)	-1.5221	-5.5119	-6.4353	-4.4898
	SDE (dB)	1.9967	0.0718	0.2754	0.7813
HATA	RMSE (dB)	10.6593	8.2441	7.9987	8.9674
	SC-RMSE (dB)	9.0571	8.1866	7.7781	8.3406
	ME (dB)	6.1322	2.1424	1.219	3.1645
	SDE (dB)	1.9967	0.0718	0.2754	0.7813
EGLI	RMSE (dB)	9.0387	10.1663	10.6778	9.9609
	SC-RMSE (dB)	7.187	10.0893	10.3828	9.2197
	ME (dB)	-3.1404	-6.3116	-7.1635	-5.5385
	SDE (dB)	2.5628	0.0921	0.3535	1.0028
ECC-33	RMSE (dB)	24.0152	20.1985	19.3447	21.1861
	SC-RMSE (dB)	22.0696	20.1286	19.0785	20.4489
	ME (dB)	22.395	18.5627	17.6545	19.5374
	SDE (dB)	2.0996	0.0761	0.2921	0.8226

**Table 6:** Error analysis for transmitter with 695.25 MHz.

MODEL		ROUTE 1	ROUTE 2	ROUTE 3	ROUTE 4	AVERAGE
KRIGING	RMSE (dB)	6.4697	12.5592	7.5838	5.7338	8.0866
	SC-RMSE (dB)	11.1662	14.7836	14.6507	11.8192	13.1049
	ME (dB)	1.27E+00	3.73E+00	1.51E+00	1.37E+00	1.97E+00
	SDE (dB)	10.4572	12.6019	14.1825	11.8331	12.2687
	Efficiency	0.9994	0.9981	0.9991	0.9995	0.999
COST 231	RMSE (dB)	9.778	8.3334	9.4373	12.8427	10.0979
	SC-RMSE (dB)	6.9789	4.7139	5.9858	7.9408	6.4049
	ME (dB)	6.9764	7.6106	5.6207	-5.9478	3.565
	SDE (dB)	4.1979	4.2889	9.0175	11.8918	7.349
HATA	RMSE (dB)	10.7071	9.4903	10.2324	12.313	10.6857
	SC-RMSE (dB)	7.7422	5.6884	6.2867	7.6562	6.8434
	ME (dB)	8.2282	8.8625	6.8725	-4.696	4.8168
	SDE (dB)	4.1979	4.2889	9.0175	11.8918	7.349
EGLI	RMSE (dB)	10.7642	6.5754	14.6407	32.4799	16.1151
	SC-RMSE (dB)	6.7287	4.3562	7.6386	19.6123	9.584
	ME (dB)	-8.3304	-4.8828	-12.1036	-29.3059	-13.6557
	SDE (dB)	5.4859	5.6047	11.7841	15.5403	9.6038
ECC-33	RMSE (dB)	22.3144	22.394	21.2087	14.0394	19.9891
	SC-RMSE (dB)	18.2817	18.042	13.2036	7.9383	14.3664
	ME (dB)	21.2402	22.1203	19.8199	8.3912	17.8929
	SDE (dB)	4.2862	4.4192	8.9869	11.6427	7.3338

**Table 7:** Error analysis for transmitter 479.25 MHz.

MODEL		ROUTE 1	ROUTE 2	ROUTE 3	ROUTE 4	AVERAGE
KRIGING	RMSE (dB)	3.1959	1.074	4.1492	6.3673	3.6966
	SC-RMSE (dB)	4.0233	1.4214	5.1372	9.9199	5.1254
	MPE (dB)	6.28E-01	2.36E-01	8.83E-01	1.33E+00	7.70E-01
	SDE (dB)	3.1514	1.1974	4.0885	9.0885	4.3815
	Efficiency	0.9999	1	0.9998	0.9995	0.9998
	Error rate (%)	0.4348	0.1644	0.6148	0.9669	0.5452
COST 231	RMSE (dB)	6.2412	8.3944	5.508	9.7283	7.468
	SC-RMSE (dB)	3.8836	7.5463	4.2706	9.1988	6.2248
	ME (dB)	-5.6794	8.3269	5.3416	7.3125	3.8254
	SDE (dB)	2.7837	0.8557	1.2879	0.7208	1.412
HATA	RMSE (dB)	4.0976	10.8813	7.9583	11.7261	8.6658
	SC-RMSE (dB)	2.6177	10.0301	6.6924	11.1297	7.6175
	ME (dB)	-3.1769	10.8293	7.844	9.815	6.3279
	SDE (dB)	2.7837	0.8557	1.2879	0.7208	1.412
EGLI	RMSE (dB)	18.0303	1.5951	3.5661	6.8575	7.5123
	SC-RMSE (dB)	14.391	0.8057	2.233	6.1053	5.8838
	ME (dB)	-17.6835	0.9087	-3.103	-2.3533	-5.5578
	SDE (dB)	3.7294	1.1464	1.7255	0.9657	1.8918
ECC-33	RMSE (dB)	9.7927	23.4923	20.5221	23.3427	19.2875
	SC-RMSE (dB)	7.1437	22.6361	19.2352	22.6496	17.9162
	ME (dB)	9.4438	23.4682	20.4779	22.4436	18.9584
	SDE (dB)	2.7863	0.8571	1.2899	0.7218	1.4138

**Table 8:** Error analysis for transmitter 559.25 MHz.

MODEL		ROUTE 1	ROUTE 2	ROUTE 3	ROUTE 4	AVERAGE
Kriging	RMSE (dB)	8.2434	10.3179	7.9186	3.2708	7.4377
	SC-RMSE (dB)	11.007	13.4184	11.7641	9.2769	11.3666
	MPE (dB)	2.22E+00	2.36E+00	1.50E+00	4.85E-01	1.64E+00
	SDE (dB)	9.8442	11.4718	10.3294	9.1806	10.2065
	Efficiency	0.9989	0.9987	0.9991	0.9998	0.9991
	Error rate (%)	1.7538	1.6627	1.1334	0.371	1.2302
COST 231	RMSE (dB)	9.778	8.3334	9.4373	12.8427	10.0979
	SC-RMSE (dB)	6.9789	4.7139	5.9858	7.9408	6.4049
	ME (dB)	6.9764	7.6106	5.6207	-5.9478	3.565
	SDE (dB)	4.1979	4.2889	9.0175	11.8918	7.349
HATA	RMSE (dB)	10.7071	9.4903	10.2324	12.313	10.6857
	SC-RMSE (dB)	7.7422	5.6884	6.2867	7.6562	6.8434
	ME (dB)	8.2282	8.8625	6.8725	-4.696	4.8168
	SDE (dB)	4.1979	4.2889	9.0175	11.8918	7.349
EGLI	RMSE (dB)	10.7642	6.5754	14.6407	32.4799	16.1151
	SC-RMSE (dB)	6.7287	4.3562	7.6386	19.6123	9.584
	ME (dB)	-8.3304	-4.8828	-12.1036	-29.3059	-13.6557
	SDE (dB)	5.4859	5.6047	11.7841	15.5403	9.6038
ECC-33	RMSE (dB)	22.3144	22.394	21.2087	14.0394	19.9891
	SC-RMSE (dB)	18.2817	18.042	13.2036	7.9383	14.3664
	ME (dB)	21.2402	22.1203	19.8199	8.3912	17.8929
	SDE (dB)	4.2862	4.4192	8.9869	11.6427	7.3338

**Table 9:** Error analysis for transmitter 615.25 MHz.

MODEL		ROUTE 1	ROUTE 2	ROUTE 3	ROUTE 4	AVERAGE
Kriging	RMSE (dB)	1.7777	11.0702	0.2351	8.5681	5.4128
	SC-RMSE (dB)	2.3358	17.5883	1.2544	9.9301	7.7772
	MPE (dB)	3.53E-01	1.92E+00	4.67E-02	1.79E+00	1.03E+00
	SDE (dB)	1.9563	15.8436	1.2798	7.2351	6.5787
	Efficiency	1	0.9984	1	0.9991	0.9994
	Error rate (%)	0.2425	1.3966	0.0323	1.2354	0.7267
COST 231	RMSE (dB)	8.4575	6.4703	10.0818	9.8673	8.7192
	SC-RMSE (dB)	7.3526	7.6445	9.1948	9.1881	8.345
	ME (dB)	8.3741	3.1626	9.9701	9.754	7.8152
	SDE (dB)	1.1176	11.8935	0.8979	0.6877	3.6492
HATA	RMSE (dB)	10.1065	7.4261	11.7287	11.5137	10.1938
	SC-RMSE (dB)	8.9976	6.9131	10.8387	10.8322	9.3954
	ME (dB)	10.0368	4.8253	11.6328	11.4168	9.4779
	SDE (dB)	1.1176	11.8935	0.8979	0.6877	3.6492
EGLI	RMSE (dB)	2.4383	13.8555	1.6716	1.5358	4.8753
	SC-RMSE (dB)	1.3918	8.9838	1.1848	1.0766	3.1593
	ME (dB)	-1.943	-11.3018	-0.1057	-0.3979	-3.4371
	SDE (dB)	1.4734	15.6797	1.1838	0.9066	4.8109
ECC-33	RMSE (dB)	23.1533	18.6074	24.7839	24.5603	22.7762
	SC-RMSE (dB)	22.0073	7.9934	23.8637	23.8555	19.43
	ME (dB)	23.1218	17.7194	24.7378	24.5151	22.5235
	SDE (dB)	1.1477	11.9557	0.922	0.7062	3.6829

## References

[bib1] Abdulrasheed I., Faruk N., Surajudeen-Bakinde N., Olawoyin L., Oloyede A., Popoola S.I. (2017). Kriging based model for path loss prediction in the VHF band. IEEE 3rd International Conference on Electro-Technology for National Development (NIGERCON).

[bib2] Abhayawardhana V., Wassell I., Crosby D., Sellars M., Brown M. (2005). Comparison of Empirical Propagation Path Loss Models for Fixed Wireless Access Systems. Vehicular Technology.

[bib3] Adebowale Q.R., Faruk N., Adewole K.S., Abdulkarim A., Olawoyin L.A., Oloyede A.A., Calafate C.T. (2021). Application of Computational Intelligence Algorithms in Radio Propagation: A Systematic Review and Metadata Analysis.

[bib4] Akinbolati A., Ajewole M. (2020). Investigation of path loss and modeling for digital terrestrial television over Nigeria. Heliyon.

[bib5] Al-Samman A.M., Rahman T.A., Azmi M.H., Al-Gailani S.A. (2018). Millimeter-wave Propagation Measurements and Models at 28 GHz and 38 GHz in a Dining Room for 5G Wireless Networks. Measurement.

[bib6] Aragón-Zavala A., Angueira P., Montalban J., Vargas-Rosales C. (2021). Radio Propagation in Terrestrial Broadcasting Television Systems: a Comprehensive Survey.

[bib7] Chen H., Chen S. (2003). A moving average based filtering system with its application to real-time QRS detection.

[bib8] Cressie N. (1988). Spatial prediction and ordinary kriging. Math. Geol..

[bib9] de Carvalho A.A., Batalha I., Alcantara M., Castro B., Barros F., Araujo J., Cavalcante G. (2021). Empirical path loss model in city-forest environment for mobile communications. J. Commun. Inf. Syst..

[bib10] Dietert J., Karger S., Rembold B. (2020). Statistical Channel Modeling Based on Raytracing Simulations.

[bib11] Egli J.J. (1957). Radio propagation above 40 MC over irregular terrain. Proc. IRE.

[bib12] Erceg V. (1999). Urban transmission loss models for mobile radio in the 900 and 1800 MHz bands. IEEE J. Sel. Area. Commun..

[bib13] Faruk N., Ayeni A., Adediran Y.A., Surajudeen–Bakinde N.T. (2013). On the study of empirical path loss models for accurate prediction of TV signal for secondary users. Progr. Electromag. Res..

[bib14] Faruk N., Ayeni A., Adediran Y. (2013). Error bounds of empirical path loss models at VHF/UHF bands in kwara state, Nigeria. IEEE EUROCON Conference.

[bib15] Faruk N., Ayeni A., Adediran Y.A., Surajudeen–Bakinde N.T. (2014). Improved path–loss model for predicting TV coverage for secondary access. Int. J. Wine Mark..

[bib16] Faruk N., Bello O., Oloyede A., Surajudeen-Bakinde N., Obiyemi O., Olawoyin L. (2017). Clutter and terrain effects on path loss in the VHF/UHF bands. IET Microw., Antennas Propag..

[bib17] Faruk N., Popoola S.I., Surajudeen-Bakinde N.T., Oloyede A.A., Abdulkarim A., Olawoyin L.A., Atayero A.A. (2019). Path loss predictions in the VHF and UHF bands within urban environments: experimental investigation of empirical, heuristics and geospatial models. IEEE Access.

[bib18] Faruk N., Surajudeen-Bakinde N.T., Abdulkarim A., Popoola S.I., Abdulkarim A., Olawoyin L.A., Atayero A.A. (2019). ANFIS model for path loss prediction in the GSM and WCDMA bands in urban area. ELEKTRIKA-J. Electr. Eng..

[bib19] González-Palacio M., Sepúlveda-Cano L., Montoya R. (2021). Simplified path loss lognormal shadow fading model versus a Support vector machine-based regressor comparison for determining reception powers in WLAN networks. International Conference on Information Technology & Systems.

[bib20] Greenwood D., Neeteson J., Draycott A. (1985). Response of potatoes to N fertilizer: dynamic model. Plant Soil.

[bib21] Hasanipanah M., Meng D., Keshtegar B., Trung N.T., Thai D.K. (2021). Nonlinear models based on enhanced Kriging interpolation for prediction of rock joint shear strength. Neural Comput. Appl..

[bib22] Hata M. (1980). Empirical formula for propagation loss in land mobile radio services. IEEE Trans. Veh. Technol..

[bib23] Igbinosa O., Okpeki U. (2019). Performance investigation of different pathloss models for a wireless communication system in Nigeria. Heliyon.

[bib24] Jimoh A., Surajudeen-Bakinde N., Faruk N., Ayeni A., Obiyemi O., Bello O.W. (2015). Performance analysis of empirical path loss models in VHF & UHF bands. Information and Communication Systems (ICICS).

[bib25] Jimoh, Surajudeen-Bakinde N., Faruk N., Bello O., Ayeni A. (2015). Clutter height variation effects on frequency dependen path loss models at UHF bands in build-up areas. Sci. Technol. Arts Res. J..

[bib26] Kram S., Nickel C., Seitz J., Patino-Studencka L., Thielecke J. (2017, October). Spatial interpolation of Wi-Fi RSS fingerprints using model-based universal kriging. 2017 Sensor Data Fusion: Trends, Solutions, Applications (SDF).

[bib27] Krige D.G. (1951). A statistical approach to some basic mine valuation problems on the Witwatersrand. J. S. Afr. Inst. Min. Metall.

[bib28] Krishnan K.V., Ganguli R. (2021). Multi-fidelity analysis and uncertainty quantification of beam vibration using co-kriging interpolation method. Appl. Math. Comput..

[bib29] Lee W.C. (1974). On the estimation of the second-order statistics of log normal fading in mobile radio environment. IEEE Trans. Commun..

[bib30] Lee W. (1998). Estimate of Local Average Power of a Mobile Radio Signal.

[bib31] Mariscotti (2011). Experimental Determination of the Propagation of Wireless Signals on Board a Cruise Ship. Measurement.

[bib32] Meng J. (2021). Raster data projection transformation based-on Kriging interpolation approximate grid algorithm. Alexandria Eng. J..

[bib33] Mezhoud N., Oussalah M., Zaatri A., Hammoudi Z. (2020). Hybrid Kriging and multilayer perceptron neural network technique for coverage prediction in cellular networks. Int. J. Parallel, Emergent Distributed Syst..

[bib34] Mitra A. (2009). Lecture notes on mobile communication. A Curriculum Development Cell Project under QIP.

[bib35] Phillips C., Ton M., Sicker D., Grunwald D. (2012). Practical radio environment mapping with geostatistics in dynamic spectrum access networks (DYSPAN). IEEE Int. Symp..

[bib36] Phillips C., Sicker D., D.G. (2013). Survey of wireless path loss prediction and coverage mapping methods. IEEE Commun. Surveys Tutorials.

[bib37] Popoola S., OSeni O. (2014). Empirical path loss models for GSM network deployment in Makurdi, Nigeria. Int. Refer. J. Eng. Sci..

[bib38] Popoola S.I., Adetiba E., Atayero A.A., Faruk N., Calafate C.T. (2018). Optimal model for path loss predictions using feed-forward neural networks. Cogent Eng..

[bib39] Popoola S.I., Atayero A.A., Faruk N. (2018). Received signal strength and local terrain profile data for radio network planning and optimization at GSM frequency bands. Data in Brief.

[bib40] Popoola S.I., Jefia A., Atayero A.A., Kingsley O., Faruk N., Oseni O.F., Abolade R.O. (2019). Determination of neural network parameters for path loss prediction in very high frequency wireless channel. IEEE Access.

[bib41] Rappaport T.S. (1996). Wireless Communications: Principles and Practice.

[bib42] Surajudeen-Bakinde N., Faruk N., Popoola S., Salman M.A., Oloyede A., Olawoyin & L.A. (2018). Path loss predictions for multi-transmitter radio propagation in VHF bands using Adaptive Neuro-Fuzzy Inference System. Eng. Sci. Technol. Int. J..

[bib43] Vicente-Serrano S.M., Saz-Sánchez M.A., Cuadrat J. (2003). Comparative analysis of interpolation methods in the middle Ebro Valley (Spain): application to annual precipitation and temperature. Clim. Res..

[bib44] Zhang L., Rodríguez-Piñeiro J., Fernández J.R., García-Naya J.A., Matolak D.W., Briso C. (2017). Propagation Modeling for Outdoor-To-Indoor and Indoor-To-Indoor Wireless Links in High-Speed Train. Measurement.

[bib45] Zheng H., Huo Y., Zhang Y., Xu R. (2018). Log-normal Fluctuation Channel Model of Short Distance. Measurement.

